# Kawasaki disease with profound systemic vascular involvements: An insightful pediatric case

**DOI:** 10.1002/ccr3.9415

**Published:** 2024-09-01

**Authors:** Naila Nadeem, Muhammad Nadeem Ahmad, Muhammad Haseeb Malik, Mallick Muhammad Zohaibuddin, Muhammad Ahmed, Faheemullah Khan, Hatem Eltaly, Uffan Zafar

**Affiliations:** ^1^ Department of Radiology The Aga Khan University Hospital Karachi Pakistan; ^2^ Department of Radiology Quaid‐e‐Azam Medical College Bahawalpur Punjab Pakistan; ^3^ Cleveland Clinic Main Campus Hospital Cleveland Ohio USA

**Keywords:** aneurysms, children, Kawasaki disease, vascular inflammatory syndrome

## Abstract

**Key Clinical Message:**

Kawasaki disease (KD), a self‐limiting vasculitis, can present with a broader spectrum of vascular involvements, necessitating early recognition and prompt management. This case exemplifies the importance of involving multiple teams on board in managing complex presentations of KD. It also highlights the importance of close monitoring for the progression of the disease spectrum as well as family education to ensure favorable outcomes. The case also emphasizes the importance of long‐term follow‐up and further research to understand the prognosis, early screening tools, and possible complications due to multi‐organ involvement in KD along with their management strategies.

**Abstract:**

Kawasaki disease (KD) is a multisystem vascular inflammatory syndrome, which predominantly affects the small and medium vessels in children within the age group of less than 5 years. The most threatened complication is the development of coronary artery aneurysms (CAAs). We present an extremely rare case of KD in a 2‐month, 21‐day‐old male infant with extensive vascular involvement, expanding the disease spectrum beyond the involvement of coronary arteries. These included aneurysmal dilatations of both internal carotid arteries, the descending aorta, bilateral multilevel intercostal arteries, coeliac artery, superior mesenteric artery, and both renal arteries. Implementing a multidisciplinary approach with early treatment via intravenous immunoglobulin (IVIG) and dexamethasone proved to be most effective in the patient's management. Despite unique challenges such as severe coronary dilation and pseudomonas sepsis during the special care, the patient was stabilized and discharged after 11 days of hospital stay, highlighting the importance of early prompt management and a centered approach to evaluate in a broader spectrum. This case emphasizes the importance of long‐term follow‐up and further research to understand the prognosis, early screening tools, and possible complications due to multi‐organ involvement in KD along with their management strategies.

## 1 INTRODUCTION

Kawasaki disease (KD) is a rare entity but one of the most common pediatric vasculitis. It predominantly affects the pediatric population, generally before the age of 5 years with coronary artery aneurysms (CAAs) being the most feared complication; however, due to the broader disease spectrum, patients can present with diverse clinical presentations.^1^ Coronary aneurysms generally develop after 2 weeks of disease onset in about 25% of cases.^2^ The standard treatment includes IV immunoglobulin and aspirin.^3^ There are no specific laboratory markers and the diagnosis is mainly based on a set of clinical criteria, featuring prolonged fever, polymorphous rash, conjunctivitis, mucosal changes, lymphadenopathy, and extremity changes.^4^ Systemic arterial aneurysm formation is a very rare entity, affecting 0.8%–2% of the cases.^5^

## 2 CASE HISTORY/EXAMINATION

We present a rare entity of extensive KD in a male infant aged 2 months and 21 days, who presented with tachycardia and tachypnea with a prolonged fever lasting 25 days, and a rash that appeared 17 days before admission. In addition, he also had a cough and congestion for 7 days. However, the major symptoms of KD, such as bulbar conjunctival injection, oral mucous membrane changes, peripheral extremity changes, and cervical lymphadenopathy, were not present. Key findings on physical examination included heart rate 110 beat/min, respiratory rate 30/min, blood pressure 91/51 mm‐Hg, oxygen saturation 99%, temperature 36.8°C, a 2/6 systolic murmur, without any added sounds and equal air entry in the chest bilaterally.

## 3 METHODS (DIFFERENTIAL DIAGNOSIS, INVESTIGATIONS, AND TREATMENT)

Initial lab workup was remarkable for raised CRP and ESR levels. The CRP was 175 mg/dL while the ESR was 78 mm/1st hour. The patient was shifted with high‐flow oxygen to the intensive care unit after the initial care in the emergency department. Based on initial echocardiography findings in ER, which had demonstrated dilatation of coronary arteries.

Focused echocardiography was subsequently performed by an expert cardiologist, which revealed severely dilated RCA at origin with aneurysm formation and distal ectasia, severely dilated LCA, severe ectasia of LAD, severely dilated left circumflex, large thrombus seen in RCA, and a small thrombus in left circumflex.

Heart failure therapy including diuretics was optimized and intravenous immunoglobulin (2 g/kg), and dexamethasone were included in the treatment plan after consulting the cardiology team. Intensive monitoring of vitals, cardiac electrolytes, and daily 12‐lead ECG were performed. The direct breastfeeding trial was tolerable, which was continued.

**FIGURE 1:**
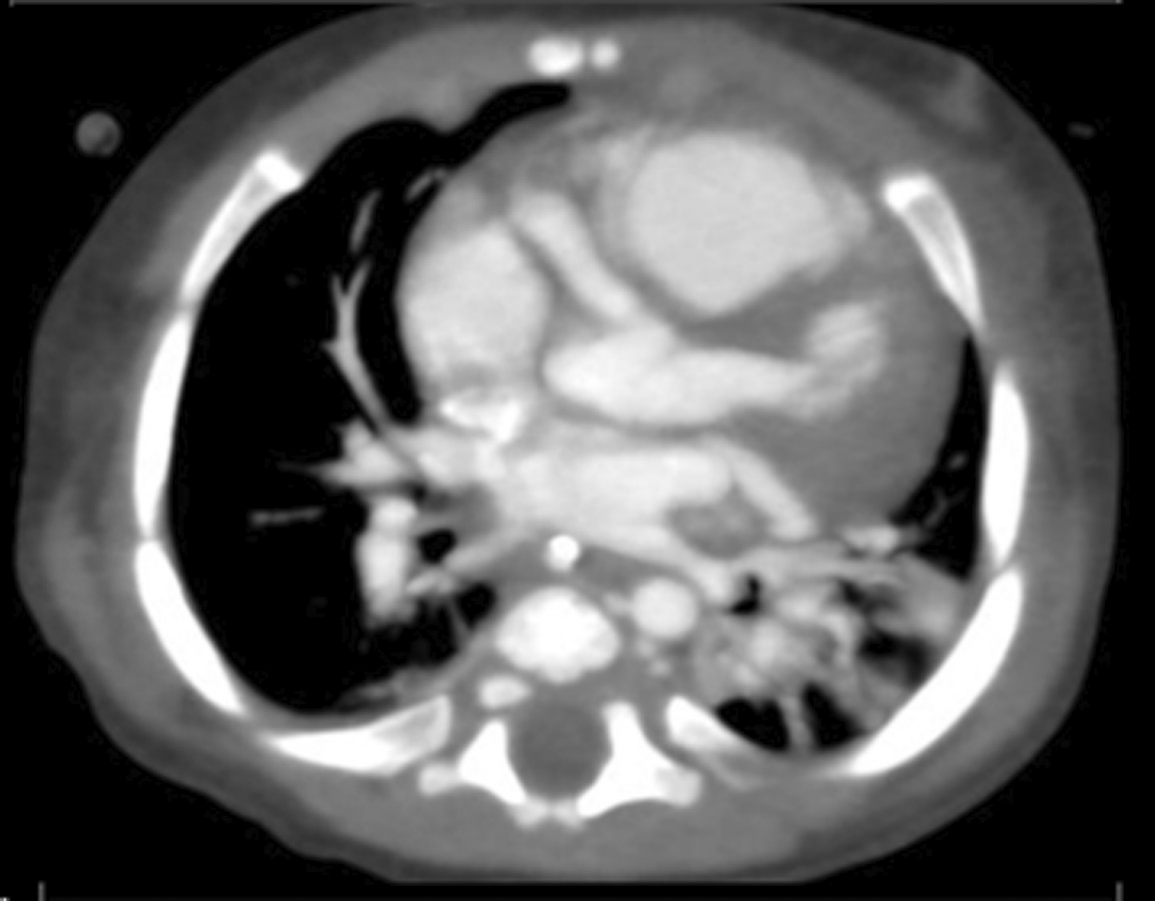
Axial CECT coronary angiogram demonstrating multiple bilateral coronary artery aneurysmal (CAA) dilatations.

**FIGURE 2:**
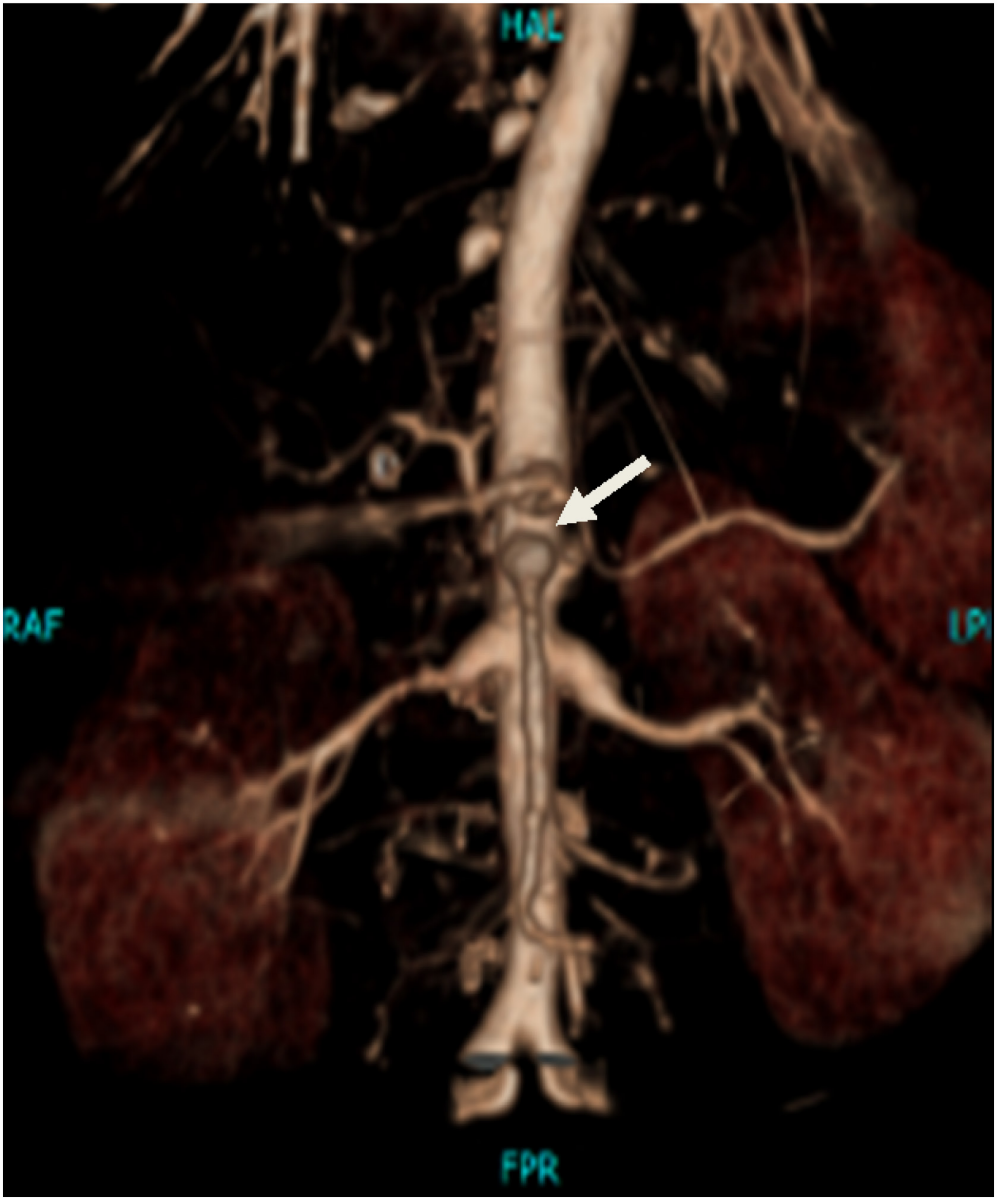
Maximum intensity projection (MIP) with coronal reconstruction and virtual cinematic rendering images, demonstrating multiple systemic arterial aneurysms in this patient, including coronary, coeliac, superior mesenteric, intercostal, and bilateral renal arteries.

**FIGURE 3:**
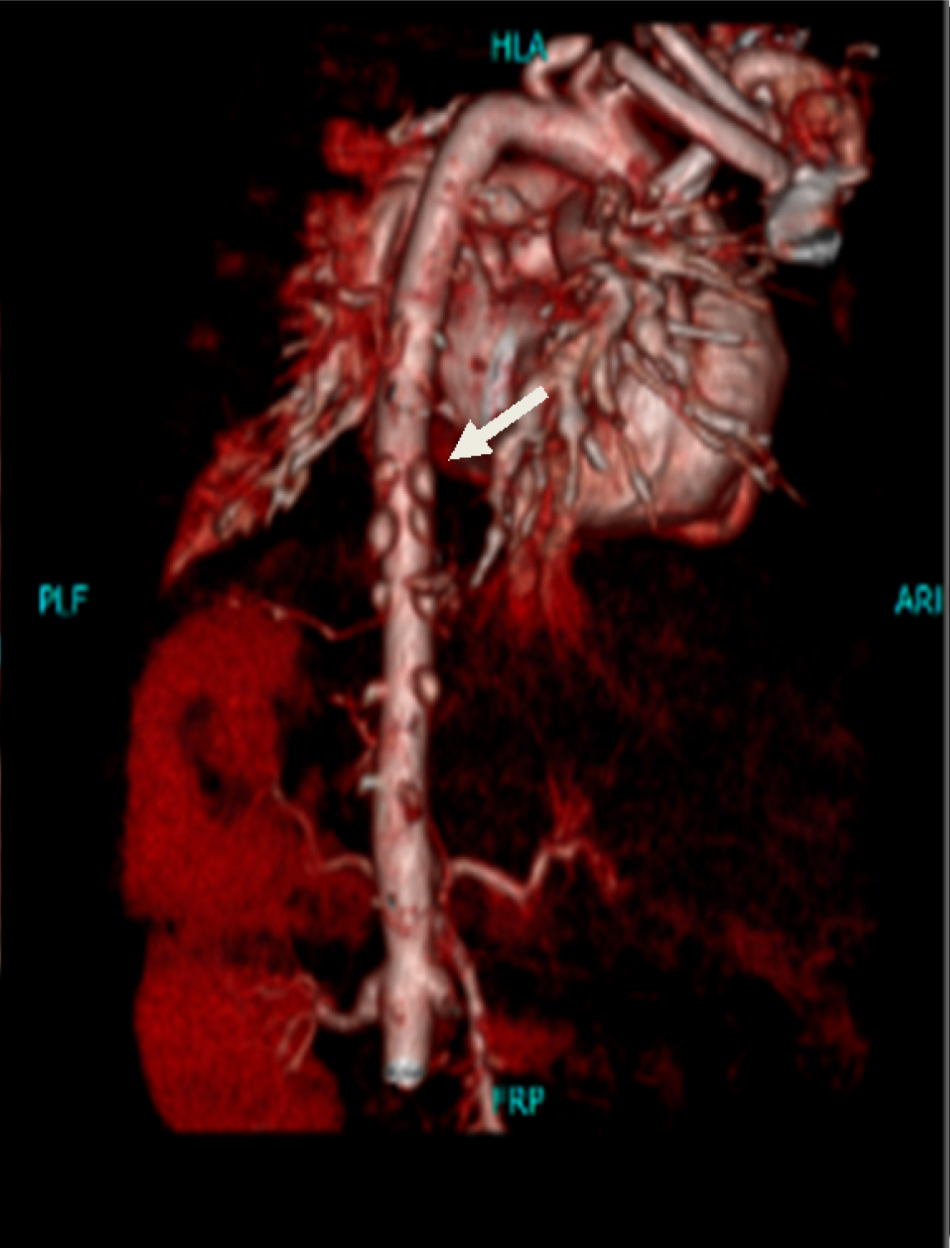
Maximum intensity projection (MIP) with coronal reconstruction and virtual cinematic rendering images, demonstrating multiple systemic arterial aneurysms in this patient, including coronary, coeliac, superior mesenteric, intercostal, and bilateral renal arteries.

**FIGURE 4:**
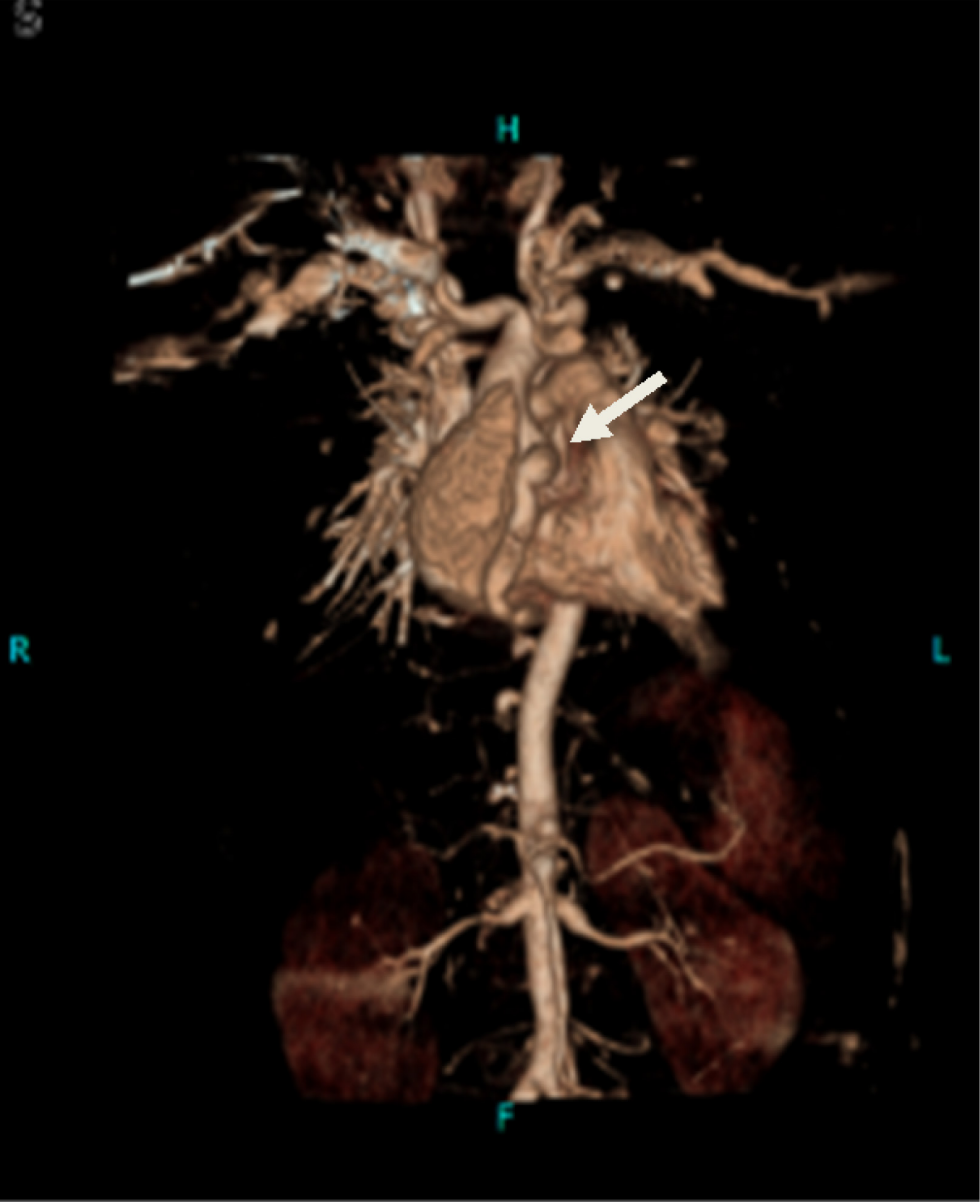
Maximum intensity projection (MIP) with coronal reconstruction and virtual cinematic rendering images, demonstrating multiple systemic arterial aneurysms in this patient, including coronary, coeliac, superior mesenteric, intercostal, and bilateral renal arteries.

As he remained stable on oral breastfeeding, he was stepped down to special care. High‐flow oxygen therapy tapered gradually. While in special care, the patient developed watery diarrhea and spikes of fever. Stool cultures, detailed report and blood cultures were sent, which revealed multidrug‐resistant pseudomonas. Antibiotic therapy was optimized with meropenem and colistin, which improved the condition within days. Diagnostic assessment through CT angiography was followed, revealing aneurysmal dilation in both internal carotid arteries, tortuous aorta with aneurysmal dilatation at the origin of bilateral intercostal arteries, coeliac, superior mesenteric, and both renal arteries, as well as aneurysmal dilatation of all coronary arteries, as shown in Figures 1, 2, 3, 4.

## 4 CONCLUSION AND RESULTS (OUTCOME AND FOLLOW UP)

An autoimmune profile was sent and the rheumatology team was taken on board. While staying in special care, a second dose of intravenous immunoglobulin (IVIG), naproxen, and immunosuppressive therapy including azathioprine and hydroxychloroquine were started. The patient remained stable and was shifted to the ward. Repeated cultures did not reveal MDR pseudomonas. The baby was later discharged after a total hospital stay of 11 days and his family was counseled for early follow‐up with infectious disease, cardiology, and hematology clinic outpatient.

## 5 DISCUSSION

Although KD is a self‐limiting disease, the main feared complication of developing coronary aneurysms can occur in up to 30% of untreated cases.^1^ This complication is one of the major causes of mortality in untreated cases, necessitating timely management. The incidence of this complication can be reduced with effective and prompt IVIG administration.^3^ Initial presentation can be variable, posing significant challenges for diagnosis, as there are no laboratory diagnostic markers and diagnosis relies mainly on classical physical signs.^4^ However, in our described case, the variability in the presentation of KD and the potential for extensive vascular spectrum, beyond the coronary arteries is key to consider. The aneurysmal dilatation in multiple arterial sites, including the bilateral internal carotid arteries, descending aorta, intercostal arteries, coeliac artery, superior mesenteric artery, bilateral renal arteries, and all coronary arteries, is a rare presentation, occurring in less than 2% of cases.^5^ This needs a systematic clinical as well as imaging approach while evaluating such patients to look for multi‐organ involvement, as the disease has the potential to show variable complexities.

While reviewing the computed tomographic study as a radiologist, one should be aware of this rare entity to look for in their checklist. This also raises a special concern for the team of infectious diseases, as our case had an added complexity in management due to MDR *Pseudomonas* sepsis. The successful management of this case involved a multidisciplinary approach, highlighting the importance of collaboration among various specialties in managing complex KD cases. IVIG, aspirin, and IV dexamethasone are the key treatments recommended for managing acute KD.^3^ However, the extensive vascular involvement in this case required vigilant monitoring and a tailored approach to address the unique challenges posed by the multiple aneurysms.

Although available literature on systemic arterial aneurysms (SAAs) in patients with KD is scarce, it has been proposed in one study that patients with larger CAAs are more likely to develop SAAs.^5^ Furthermore, it has been observed that screening for SAAs after 3 months of the onset of KD may underestimate the incidence of SAA.^6^

However, since screening for SAAs by angiography may not always be feasible, researchers must explore factors that point toward the development of SAAs and narrow down the candidates who should undergo screening for SAAs. A study performed to evaluate SAAs in KD^7^ found that only 17.4% showed SAA‐related abnormalities on physical examination, such as a weakened radial pulse or pulsatile axillary masses. The majority (95.7%) of these SAAs were detected by MRA rather than peripheral angiography, while all the SAAs had concomitant giant or medium CAAs. Other positive relations of SAAs found in this study were a younger age of onset (5 months) and a longer duration of fever (12 days), which were also seen in the present case. However, the systemic arteries mostly involved in this study differed from the present case, including the axillary, common iliac, brachial, internal iliac, and subclavian arteries. These findings suggest that the possibility of SAAs in KD must be ruled out in patients who present at a younger age or have a fever for a longer duration even with few major symptoms of KD. It may be useful that these patients are screened for SAAs.

This case emphasizes acute as well as long‐term prospective plans for management. It emphasizes the need for long‐term follow‐up and monitoring of patients with KD, especially those with extensive vascular involvement, to detect and manage potential complications such as aneurysm progression or thrombosis and multi‐organ involvement.^5^ Previously, there have been case reports of young patients presenting with calcified coronary aneurysms, highlighting the importance of screening patients for the progression of the disease.^8^ This also highlights the importance of family education regarding disease progression, prognosis, and regular follow‐ups. The limitation of our case is the lack of a long‐term follow‐up date, which would provide valuable insights regarding the progression of other possible vascular involvement and multi‐organ complications. Further research is needed in these patients with extensive vascular involvement, which would explain and redefine the standards of approach toward such patients in the long run.

## AUTHOR CONTRIBUTIONS

**Naila Nadeem:** Conceptualization; data curation; investigation; project administration; writing – original draft. **Muhammad Nadeem Ahmad:** Conceptualization; writing – original draft. **Muhammad Haseeb Malik:** Conceptualization; data curation; supervision. **Mallick Muhammad Zohaibuddin:** Conceptualization; data curation; supervision. **Muhammad Ahmed:** Conceptualization; data curation; validation. **Faheemullah Khan:** Conceptualization; supervision; validation. **Hatem Eltaly:** Conceptualization; data curation; investigation; validation; writing – original draft. **Uffan Zafar:** Conceptualization; data curation; investigation; project administration; writing – original draft.

## FUNDING INFORMATION

No funding was received to assist with the preparation of this manuscript.

## CONFLICT OF INTEREST STATEMENT

The authors declare that they have no known competing financial interests or personal relationships that could have appeared to influence the work reported in this paper.

## ETHICS STATEMENT

Not applicable.

## CONSENT

Written informed consent was obtained from the patient's parents to publish this report in accordance with journal's patient consent policy.

## Data Availability

The data supporting the findings of this study are available from the corresponding author upon reasonable request.
